# Analysis of the *F2LR3* (PAR4) Single Nucleotide Polymorphism (*rs773902*) in an Indigenous Australian Population

**DOI:** 10.3389/fgene.2020.00432

**Published:** 2020-04-30

**Authors:** Dian Ningtyas, Russell J. Thomson, Volga Tarlac, Shivashankar H. Nagaraj, Wendy Hoy, John D. Mathews, Simon J. Foote, Elizabeth E. Gardiner, Justin R. Hamilton, Brendan J. McMorran

**Affiliations:** ^1^Department of Immunology and Infectious Disease, The John Curtin School of Medical Research, The Australian National University, Canberra, ACT, Australia; ^2^Centre for Research in Mathematics and Data Science, School of Computer, Data and Mathematical Sciences, Western Sydney University, Parramatta, NSW, Australia; ^3^Australian Center for Blood Diseases, Faculty of Medicine, Nursing and Health Sciences, Monash University, Melbourne, VIC, Australia; ^4^Institute of Health and Biomedical Innovation, School of Biomedical Sciences, Queensland University of Technology, Brisbane, QLD, Australia; ^5^Translational Research Institute, Brisbane, QLD, Australia; ^6^Centre for Chronic Disease, Faculty of Health, The University of Queensland, Brisbane, QLD, Australia; ^7^Centre for Epidemiology and Biostatistics, Melbourne School of Population and Global Health, University of Melbourne, Melbourne, VIC, Australia; ^8^Menzies School of Health Research, Darwin, NT, Australia

**Keywords:** protease activated receptor 4, *rs773902*, renal disease, Australian Aboriginal and Torres Strait Islanders, indigenous genetics

## Abstract

The *F2RL3* gene encoding protease activated receptor 4 (PAR4) contains a single nucleotide variant, *rs773902*, that is functional. The resulting PAR4 variants, Thr120, and Ala120, are known to differently affect platelet reactivity to thrombin. Significant population differences in the frequency of the allele indicate it may be an important determinant in the ethnic differences that exist in thrombosis and hemostasis, and for patient outcomes to PAR antagonist anti-platelet therapies. Here we determined the frequency of *rs773902* in an Indigenous Australian group comprising 467 individuals from the Tiwi Islands. These people experience high rates of renal disease that may be related to platelet and PAR4 function and are potential recipients of PAR-antagonist treatments. The *rs773902* minor allele frequency (Thr120) in the Tiwi Islanders was 0.32, which is similar to European and Asian groups and substantially lower than Melanesians and some African groups. Logistic regression and allele distortion testing revealed no significant associations between the variant and several markers of renal function, as well as blood glucose and blood pressure. These findings suggest that *rs773902* is not an important determinant for renal disease in this Indigenous Australian group. However, the relationships between *rs773902* genotype and platelet and drug responsiveness in the Tiwi, and the allele frequency in other Indigenous Australian groups should be evaluated.

## Introduction

Platelet activation by thrombin during hemostasis and thrombosis is mediated through two therapeutically tractable protease-activated receptors, PAR1 and PAR4. The gene encoding PAR4 (*F2RL3*) contains a functionally important single nucleotide polymorphism (*rs773902*) expressing either an Ala120 or Thr120 variant receptor. The Thr120 variant is associated with higher aggregation and Ca^2+^ flux induced by PAR4 agonists in isolated platelets (Edelstein et al., 2014; Whitley et al., 2018), and greater *ex vivo* thrombosis formation (Tourdot et al., 2018). The allele frequencies for this variant differ according to ethnicity, with the Thr120 variant relatively common in some African populations (up to 68%) and less common in Asians and Europeans (19–29%; Edelstein et al., 2014; Heenkenda et al., 2018). Previous studies comparing PAR4-dependent platelet functions in African and “white” Americans indicated distinct differences (Edelstein et al., 2013; Tourdot et al., 2014), which in other studies have been directly associated with Ala120/Thr120 variant status (Edelstein et al., 2014). The variant may also be clinically important. One study has shown a greater risk of thrombosis amongst carriers of the Thr120 in a general American population (Whitley et al., 2018). In addition, the relative benefits and risks of using the PAR1 antagonist vorapaxar may vary according to the PAR4 sequence variant, with a suggestion of lower bleeding rates in Thr120-expressing individuals treated with vorapaxar than in Ala120-expressing individuals (Tricoci et al., 2018). Furthermore, PAR4 antagonists are in clinical development (French and Hamilton, 2017; Wong et al., 2017) and the impact of the *rs773902* PAR4 SNP on the efficacy and safety of these agents remains unexplored.

Indigenous Australians, who represent a distinct population with ancestral and genetic histories that predate all cultures outside of Africa, suffer very high burdens of cardiovascular disease, stroke, diabetes, and chronic kidney disease (CKD; Australian Institute of Health and Welfare, 2016). For example, diabetes deaths in Indigenous Australians are increased 15-fold, cardiovascular deaths by 3–6 fold (Hoy, 1996), and rates of treated end-stage renal disease are 20 times more than in non-Indigenous Australians (Spencer et al., 1998). Studies in these people have also reported familial aggregation amongst cases of CKD (Van Buynder et al., 1993), and significant genetic heritability and genetic associations with markers of renal function (Duffy et al., 2016; Thomson et al., 2019). Both platelets and PAR4 have been implicated in kidney function and CKD pathogenesis (Lambert, 2016; Madhusudhan et al., 2016; Palygin et al., 2016), and renal disease patients are prone to bleeding and thrombotic events (Saheb Sharif-Askari et al., 2017). However, the allele frequencies of *rs773902* have not been investigated in Indigenous Australians. Therefore, we determined the genotype and allele frequencies of *rs773902* in an Indigenous Australian group native to The Tiwi Islands and analyzed the associations between this variant and markers of renal disease.

## Methods

### Study Participants

The study participants all lived on The Tiwi Islands, located off the northern coast of Australia in the Arafura Sea. Tiwi Islanders exhibit a distinct genetic ancestry compared to other ethnicities (Thomson et al., 2019), and are considered most closely related to mainland Indigenous Australians (Tiwi Land Council, 2018). The current total population is approximately 2,500 individuals (ABS, 2016). All the participants were self-identifying Tiwi Islanders. They were recruited as clinic outpatients and consented to collection of blood and urine samples, primarily for characterization of renal function and DNA for genetic studies. The numbers of participants, their age ranges and the respective clinical measures used in the study are shown in Table 1. None of the participants were experiencing renal failure or receiving renal replacement therapy at the time the study was performed. A previous analysis of a separate cohort of self-identifying Tiwi Islanders (*n* = 73 individuals) indicated low admixture with other populations, including Europeans (Thomson et al., 2019). The study received the full support of The Tiwi Island Land Council and was approved by the human research ethics committees of The Northern Territory Department of Health (2012–1767), The Australian National University (2014–663), The University of Queensland (2012001146), and The University of Tasmania (H0012832).

**TABLE 1:** Sample characteristics.

**Tiwi group**	**Sex**	**Age (year)**	**ACR (g/mol)**	**Systolic BP (mmHg)**	**Diastolic BP (mmHg)**
		**median (range)**	**median (range)**	**median (range)**	**median (range)**
All samples	228F, 239M	39 (17–75)	2.3 (0.1–1755)*	113 (71–187)**	74 (48–124)**
CKD unaffected	30F, 30M	37 (30–58)	0.5 (0.1–0.9)	121 (71–187)	77 (51–115)
CKD affected	30F, 30M	43 (19–60)	92.2 (30.2–1755)	107 (85–134)	71 (56–91)

*Data calculated from 455 individuals. **Data calculated from 466 individuals.

### Genotyping and Allele Frequency Analysis

A total of 467 participants were successfully genotyped for the *rs773902* allele. Genotyping was performed on 2.5 ng of genomic DNA prepared from blood samples using a TaqMan assay and 7900HT Fast Real-Time PCR System (Applied Biosystems), and standard protocols. A subset of randomly chosen samples (*n* = 70) subjected to replicate genotyping were in 100% agreement. Chi-square test for genotype frequency distortions from Hardy Weinberg equilibrium were performed using the WPcalc online tool (Wow-Company).

### Clinical Measures

Blood and urine specimens were non-fasting and collected without regard for time of day. Serum was separated immediately after clotting, within 20 min in all cases. All specimens were then refrigerated until transfer to the pathology laboratory by air, within 24 h. Albumin (mass) and creatinine (molar) concentrations were analyzed in the spot urine sample and an albumin/creatinine ratio (ACR) calculated (g/mol). The estimated glomerular filtration rate (eGFR, mL/min/1.73 m^2^) was determined from serum creatinine levels using the MDRD Study equation (Levey et al., 1999). Blood glucose levels were determined using standard diagnostic laboratory assay. Blood and leukocyte positive urine samples were identified using urine test strips (Bayer Multistix 10 SG Reagent Urinalysis Test Strips), defined as small, moderate or large, and ≥1+, respectively. Systolic and diastolic blood pressure (BP) were measured by a single observer using a mercury sphygmomanometer. The median and range of the ACR, systolic and diastolic BP measures amongst the study participants are provided in Table 1.

### Association Studies

Logistic regression was used to estimate the odds ratio between disease markers and the *rs773902* Thr120 allele using a dose dependent model, and adjustments for age and sex. The markers used were micro- and macroalbuminuria (ACR > 3.5 g/mol and ACR > 30 g/mol; available for 455 participants), eGFR < 90 mL/min/1.73 m^2^ (467 participants), blood positive urine (dipstick result: large, moderate and small; 454 participants), leukocyte positive urine (dipstick result: ≥1+; 454 participants), hypertension (systolic blood BP > 140 and diastolic BP > 90; 466 participants), and random blood glucose (>12.5 mmol/L; 460 participants). The association between renal disease and the *rs773902* Thr120 allele was also examined with a logistic regression model using a subset of 60 CKD affected and 60 unaffected individuals (Table 1). The 60 CKD affected individuals were the youngest 30 males and 30 females of the cohort with ACR ≥ 34 g/mol and any eGFR value. The 60 unaffected individuals were the oldest 30 males and 30 females of the cohort with ACR ≤ 3.4 g/mol and eGFR ≥ 90 mL/min/1.73 m^2^. These groupings were assigned in consultation with renal physician Prof. Matthew Jose, University of Tasmania. Selection according to age was important because of the high frequency of early-onset renal disease in the Tiwi Islander population (Hoy et al., 1998), and was applied to minimize inclusion of healthy younger aged individuals in the unaffected group (who may go to develop CKD at a later age) and maximize inclusion of those with early-onset disease in the affected group. The age distributions of each group are presented in the Supplementary Figure S1. Linear regression modeling was used to examine the relationship between the *rs773902* Thr120 allele and the response variables, ACR, systolic and diastolic BP. The response variable was log transformed for all models and the beta coefficients were exponentiated to provide response ratios. All models were adjusted for age and sex, and *p*-value thresholds of significance were adjusted for multiple testing using the Bonferroni method.

## Results and Discussion

The allele frequencies for *rs773902* determined for the entire cohort satisfied Hardy-Weinberg equilibrium and the minor allele frequency (MAF), encoding Thr120, was 0.32 (Table 2). Logistic regression analyses indicated no significant relationships between the Thr120 allele and indicators of abnormal renal function, including microalbuminuria, macroalbuminuria, eGFR < 90 mL/min/1.73 m^2^, and blood-positive and leukocyte-positive urine samples, or hypertension and random blood glucose concentration. A separate analysis of the allele frequencies in individuals categorized as CKD affected versus unaffected (*n* = 60/60) also revealed no significant relationship (Table 3). Analyses using linear regression modeling likewise did not detect any significant associations with ACR or BP within the whole study cohort (Table 4). The small sample sizes in our study may be a limiting factor for detecting statistically significant differences; by comparison, the seminal study reporting significant associations between *rs773902* and stroke risk included more than 6000 individuals (Whitley et al., 2018). Due to an absence of data in our cohort, it was not possible to directly test if cardiovascular disease or thrombosis were associated with *rs773902*.

**TABLE 2:** *rs773902* genotype and allele frequencies in all study participants.

**Genotype count**			**Total count**	**Allele frequency**		HW Chi-square*
**Ala120/Ala120**	**Thr120/Ala120**	**Thr120/Thr120**		**Ala120**	**Thr120**	
216	206	45	467	0.68	0.32	0.17

*Chi-square statistic for Hardy-Weinberg (HW) genotype equilibrium; threshold for significance is Chi-square >5.99 (2 degrees of freedom, and alpha = 0.05).

**TABLE 3:** The association between disease markers and the *rs773902* Thr120 allele.

**Disease**	**Sample size (disease, control)**	Odds ratio* (95% CI)	*p-*value**
Microalbuminuria (ACR > 3.5 g/mol)	194, 261	1.18 (0.87–1.6)	0.28
Macroalbuminuria (ACR > 30 g/mol)	78, 377	1.11 (0.77–1.61)	0.56
eGFR < 90 mL/min/1.73 m^2^	137, 328	0.83 (0.59–1.17)	0.29
Blood positive urine (dipstick result: large, moderate, and small)	165, 289	1.31 (0.98–1.78)	0.073
Leukocyte positive urine (dipstick result: ≥1+)	114, 340	0.8 (0.57–1.13)	0.21
Hypertension (systolic BP>, <140 mmHg)	21, 445	1.48 (0.75–2.89)	0.25
Hypertension (diastolic BP>, <90 mmHg)	26, 440	1.76 (0.98–3.16)	0.057
Random blood glucose (>12.5 mmol/L)	38, 422	0.75 (0.42–1.29)	0.31
Renal disease (CKD, unaffected)	60, 60	1.39 (0.79–2.48)	0.26

CI, Confidence interval; *odds ratio of disease for each extra Thr120 allele. **Threshold for significance after correction for multiple testing is *p* < 0.0006.

**TABLE 4:** The association between disease endophenotypes and the *rs773902* Thr120 allele.

**Endophenotype**	**Sample Size**	**β value**	**SE (β)**	Response Ratio* (95% CI)	*p*-value**
ACR	455	0.18	0.14	1.2 (0.92–1.57)	0.18
Systolic blood pressure	466	0.013	0.0083	1.01 (0.996–1.03)	0.13
Diastolic blood pressure	466	0.0066	0.0087	1.01 (0.989–1.03)	0.49

SE, Standard error; CI, Confidence interval; *Response ratio represents the ratio between the ACR or BP of participants for each extra Thr120 allele. **Threshold for significance after correction for multiple testing is *p* < 0.017.

The MAF for *rs773902* measured in the Tiwi Islanders (0.32) was slightly higher than the 0.19–0.29 range observed in various Asian and European groups, but substantially lower than the 0.57–0.63 range reported in some African groups [(Edelstein et al., 2014; Heenkenda et al., 2018); ExAc and gnomAD data sets]. Notably, the Tiwi Islander frequency was also lower than Melanesian and Papua New Guinean frequencies (0.55, Human Genome Diversity Project data set), who are considered to have a close genetic relationship to Indigenous Australians (McEvoy et al., 2010) – although it should also be noted the Melanesian allele data are from only 35 individuals. The method of participant recruitment for this study was not randomized across the population, however, the relatively large sample size (representing approximately 20% of the entire Tiwi Island population) and the similarities in disease burdens to other community-wide based surveys of the Tiwi (Hoy et al., 1998; Hoy et al., 2017) indicate the *rs773902* allele frequencies reported here are an accurate reflection of the Tiwi Islander people.

The high burden of diseases related to platelet function in Indigenous Australians and the concomitant use of anti-platelet therapies should motivate further investigation to understand the functional effects of the variant in these populations. It will also be of interest to determine if the PAR4 *rs773902* allele frequency found in the Tiwi is representative of other Indigenous Australian groups since many have existed in relative isolation from each other for several 1000 years.

## Data Availability Statement

The datasets for this article are not publicly available due to privacy concerns for the study participants and the indigenous community involved. Requests to access the datasets should be directed to the corresponding author and/or the Tiwi Land Council, admin@tiwilandcouncil.com.

## Ethics Statement

The studies involving human participants were reviewed and approved by the human research ethics committees of The Northern Territory Department of Health (2012-1767), The Australian National University (2014-663), The University of Queensland (2012001146), and The University of Tasmania (H0012832). The patients/participants provided their written informed consent to participate in this study. The study received the full support of The Tiwi Island Land Council.

## Author Contributions

BM, RT, and JH wrote the manuscript. BM, EG, and JH conceived the study. BM, DN, and VT carried out the genotyping. RT and SN carried out the statistical analyses. JM, WH, and SF founded the Tiwi kidney disease and genetic studies, were instrumental in working with the Tiwi community and Tiwi Land Council, and contributed clinical knowledge. All authors read and approved the final manuscript.

## Conflict of Interest

The authors declare that the research was conducted in the absence of any commercial or financial relationships that could be construed as a potential conflict of interest.
