# Supplementary material for: Analysis of the F2LR3 (PAR4) Single Nucleotide Polymorphism (rs773902) in an Indigenous Australian Population
Source: Front Genet. 2020 Apr 30;11:432. doi: 10.3389/fgene.2020.00432 (PMC7204273; doi:10.3389/fgene.2020.00432)
Supplement: Supplementary file 1 [file Data_Sheet_1.PDF]

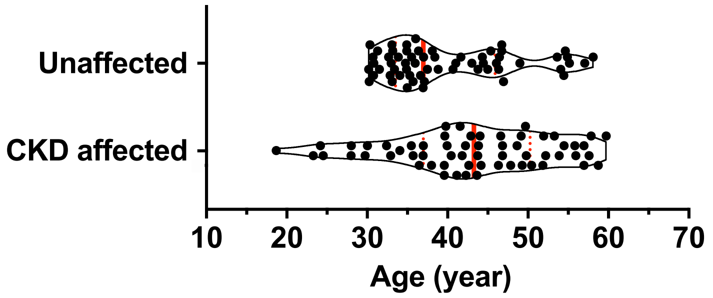

Supplementary Figure 1. Violin plot showing age distributions, medians (red solid lines) and quartiles (red dotted lines) for the chronic kidney disease (CKD) affected and unaffected groups.
